# Team performance training for medical students: Low vs high fidelity simulation

**DOI:** 10.1016/j.amsu.2020.05.042

**Published:** 2020-05-29

**Authors:** Marios Nicolaides, Efthymia Theodorou, Elif Iliria Emin, Iakovos Theodoulou, Nikolai Andersen, Nikolaos Lymperopoulos, Funlayo Odejinmi, Dilek Kitapcioglu, Mehmet Emin Aksoy, Apostolos Papalois, Michail Sideris

**Affiliations:** a Barts and the London School of Medicine and Dentistry, Queen Mary University of London, London, United Kingdom; b Faculty of Life Sciences and Medicine, King's College London, London, United Kingdom; c PPA-International Medical, Denmark; d Royal Marsden Hospital NHS Trust London, London, UK; e Department of Obstetrics and Gynaecology, Whipps Cross University Hospital, London, United Kingdom; f Center of Advanced Simulation and Education, Acibadem Mehmet Ali Aydinlar University, Istanbul, Turkey; g European University Cyprus, Nicosia, Cyprus; h Experimental, Educational and Research Centre Elpen, Athens, Greece; i Blizard Institute of Cell and Molecular Science, Queen Mary University of London, London, United Kingdom

**Keywords:** Education and training, Trauma management, Accident and emergency medicine, Medical education and training

## Abstract

**Objectives:**

The aim of this study is to evaluate a simulation-based team performance course for medical students and compare its low- and high-fidelity components.

**Study design:**

This is a prospective crossover observational study. Groups participated in one low- and one high-fidelity session twice. Low-fidelity scenarios included management of an emergency case on a simulated-patient, whereas high-fidelity scenarios constituted of multiple-trauma cases where simulated-patients wore a hyper-realistic suit. Team performance was assessed objectively, using the TEAM™ tool, and subjectively using questionnaires. Questionnaires were also used to assess presence levels, stress levels and evaluate the course.

**Results:**

Participants’ team performance was higher in the low-fidelity intervention as assessed by the TEAM™ tool. An overall mean increase in self-assessed confidence towards non-technical skills attitudes was noted after the course, however there was no difference in self-assessed performance between the two interventions. Both reported mean stress and presence levels were higher for the high-fidelity module. Evaluation scores for all individual items of the questionnaire were ≥4.60 in both NTS modules. Students have assessed the high-fidelity module higher (4.88 out of 5, SD = 0.29) compared to low-fidelity module (4.74 out of 5, SD = 0.67).

**Conclusions:**

Both the low- and high-fidelity interventions demonstrated an improvement in team performance of the attending medical students. The high-fidelity intervention was more realistic, yet more stressful. Furthermore, it proved to be superior in harvesting leadership, teamwork and task management skills. Both modules were evaluated highly by the students, however, future research should address retention of the taught skills and adaptability of such interventions.

## Highlights

• The high-fidelity intervention was superior in harvesting leadership, teamwork and task management skills, yet more stressful.
• We set the group to develop a novel group of team-based OSCE performance.
• The next step is to adapt such taught skills to different specialties and implement initiatives in the medical school curricula.

## Strengths and limitations of this study

• Our described interventions were successful in improving medical students' team performance.
• The high-fidelity intervention was more realistic, and superior in harvesting leadership, teamwork and task management skills, yet more stressful.
• Data collection was done at a single institution with a small sample size.
• Future work should focus on adaptability of taught skills to different specialties and medical topics, as well as the potential to implement such training intervention to medical school curricula.

## 1 Introduction

As of 2012, globally 312.9 million people undergo surgery each year; of this number, 50 million are estimated to suffer from complications of surgery [1], half of which occur in the operating theatre and only a tenth being attributed to technical errors [2]. Notably, such findings have followed the emergence of prominent reports, such as ‘To Err is Human’ and ‘An organisation with memory’, declaring a considerable mortality rate due to preventable medical errors, both in the US and the UK. Additionally, several root cause analyses have come forward identifying non-technical skills deficiencies as significant contributors to surgical errors [[3], [4], [5]], attracting the medical community's attention to the importance of such skills and questioning the integrity of the training system in place. In response, professional organisations such as The Royal College of Surgeons of England and Edinburgh have introduced non-technical skills courses into surgical training and have developed rating systems for their evaluation [6,7].

Non-technical skills (NTS) in medical education can be defined as a cohesion of ‘soft skills’, allowing doctors to self-evolve as part of a ‘learning organization’ capable of adapting in volatile environments [8]. Although the importance of NTS training has indeed been recognised in the past decade by professional organisations, efforts to standardise and promote such teaching modalities have been directed primarily at the post-graduate training stage, overlooking medical undergraduates [[9], [10], [11]]. These shortcomings, in addition to the concept of dehumanization, supporting that medical students become progressively detached from patients [8], signals the need for a more structured and unified training approach to be integrated in modern medical school curricula.

NTS were initially developed by the aviation industry in the late 1970s by retrospectively analysing accidents and identifying deficiencies in team performance and other non-practical competencies [12]. They later found their way into medicine via anaesthetics, surgery and emergency medicine; specialties which by foundation require rapid decision making and excellent teamwork, leadership, communication and task management skills [10,13]. Taking this into consideration, NTS training efforts at the undergraduate level should utilise emergency and surgical scenarios to yield maximum profits. Additionally, they should make use of simulation-based approaches, which are becoming increasingly favourable in surgical education [14]. Even though virtual and augmented reality simulation training has attracted the lion's share of funding and attention, the use of simulated patients remains the most widely used and validated modality in undergraduate education [14]. This can be mostly attributed to its ability to nurture skills and attitudes effectively, whilst also maintaining patient safety, being cost effective, adaptable and easy to use [[15], [16], [17]]. Even though a limited number of studies have attempted to compared low- and high-fidelity simulators, a consensus is yet to be reached on the ideal fidelity level and, most notably, all previous studies involved practical skills only [[18], [19], [20]].

The ‘Essential Skills in the Management of Surgical Cases’ (ESMSC) course is a three-day program for medical students. It combines high-fidelity and low-fidelity simulation-based learning (SBL), with applied and basic science case-based workshops, and non-technical skills modules [21]. The aim of this study is to evaluate a simulation-based team performance course for medical students and compare its low- and high-fidelity components.

## 2 Methods

### 2.1 Course concept

This is a prospective crossover observational study. The NTS modules described in this study are part of a 3-day program for medical students, the ESMSC, taking place bi-annually in Europe. NTS is one of the four cores of the curriculum, Cores Integrated for Research – Ci4R, previously described in detail [21]. ESMSC Marathon Course has met directive 63/2010, PD 56/April 2013 declaration, according to local policy. The license reference number is 884 28/4/2015, MS, AP et al.

### 2.2 Patient and public involvement

This study directly involves medical students and not patients; the research question was developed on the basis of designing a novel curriculum module to train the future generation of doctors. The outcome measures were defined by a validated tool (TEAM™ tool) and mainly refer to non-technical skills performance. Dissemination of the results took place via structured feedback sessions as well as on publication of this manuscript. Although patients were not directly involved in this study, the study aims to optimise communication of junior doctors and hence indirectly benefit patients.

### 2.3 Student recruitment and faculty selection

Medical students were invited to apply online if the eligibility criteria were met. These included completion of the medical pre-clinical studies and proficiency in English. Eligible candidates were offered a place upon competitive assessment of their personal statement and curriculum vitae. Faculty were invited to attend upon careful consideration by an expert panel (AP, MS) and were required to have expertise in the taught topic and previous experience in simulated-based teaching.

### 2.4 Module design

Delegates were allocated into groups of five based on their country of study. Prior to the NTS module, students went through teaching on a systematic method for managing acutely ill patients, the ABCDE approach [22]. Each group participated in one low- and one high-fidelity NTS session twice; the first run was considered a mock and the second the ‘actual’. Groups were given a scenario and had 20 min to stabilise a simulated patient-actor. The modulator would prompt or aid the participants during the mock attempt. After the mock, a dedicated 20-min slot was allocated for case-based discussion, non-technical skill teaching and constructive feedback. Sufficient time was allowed for the participants to rest and reflect before the actual attempt.

**Fig. 1:**
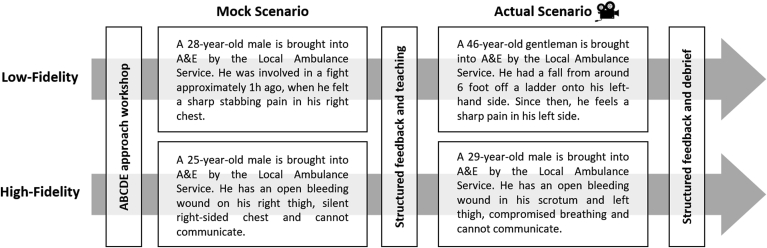
Student-groups went through one mock and one actual scenario in both low- and high-fidelity settings.

**Fig. 2:**
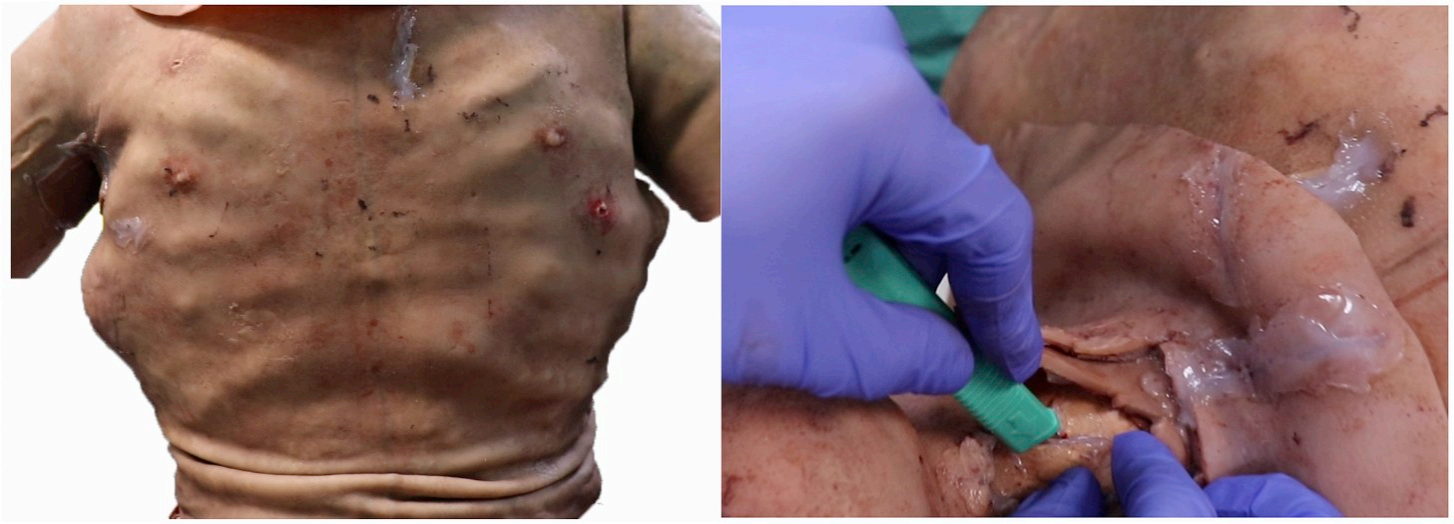
Hyper-realistic training suit by PPA-International Medical a) Suit for multi-trauma cases on a simulated-patient b) Surgical tracheostomy performed by a student during the high-fidelity scenario.

The scenarios were developed by the authors to challenge students at the undergraduate medical level without requiring any specialist knowledge (Fig. 1). Low-fidelity scenarios included management of an emergency case on a simulated-patient; liver laceration or spleen rupture. The actor was debriefed in advance on how to respond to questions and examination. Students could ask the session modulator for any patient observations (e.g. heart rate) or investigation outcomes (e.g. x-ray). Information that was obtained from the history, examination and investigations was used to conclude on a probable diagnosis and management plan for stabilising the patient. High-fidelity scenarios constituted of multiple-trauma cases where simulated-patients wore a hyper-realistic suit (Fig. 2). The specialised suit allowed for a range of practical skills to be performed, including a surgical tracheostomy, chest drain insertion and bleeding control (Fig. 2). In addition, a monitor would display the vital observations of the patient; which were adjusted wirelessly by the group modulator using a tablet.

The aim of the groups in all scenarios was to stabilise the patient, work and communicate effectively within the team. Practical skills performed, including examination and surgical interventions, were not assessed.

### 2.5 Assessment

Questionnaires were filled in by participants at the beginning of the course and after both the low- and high-fidelity scenarios. A concoction of binary-like (yes/no) and 5-point Likert-scale questions were used. The aim of the pre-course questionnaire was to collect demographic information, as well as scoring participants' attitude towards non-technical skills. Assessed attitudes included listening attentively and asking questions for clarification. The aim of the post-scenario questionnaires was to assess any changes in attitude towards non-technical skills, evaluate perceived stress and ‘presence’ in the simulated environment, as well as utilised the training evaluation inventory (TEI) to appraise subjective enjoyment, perceived usefulness, difficulty and attitude towards training.

NTS performance was assessed using the Team Emergency Assessment Measure (TEAM) tool [23] by two independent assessors (MN, ET), following thorough review of the video recordings. The tool aims to assess leadership, teamwork and task-management. Performance was only assessed during the actual scenarios of the low- and high-fidelity modules.

### 2.6 Data collection and statistical analysis

Filled-in printed questionnaires were scanned and exported to a digital spreadsheet, matched into their respective student group (A-E) and categorised as pre-intervention, post-low-fidelity and post-high-fidelity. These were then analysed using IBM SPSS Statistics for Windows, Version 25.

A paired-samples *t*-test was used by default to determine whether there was a statistically significant mean difference between the questionnaires before participants having any intervention compared to after attending the low- and high-fidelity NTS modules of the course. Outlies that were more than 1.5 box-lengths from the edge of the box were detected in a boxplot and the assumption of normality was assessed by Shapiro-Wilk's test (*p* > 0.05). A Wilcoxon signed-rank test was used instead when deemed necessary to draw or confirm any conclusions made. A one-way repeated measures MANOVA was carried out for multivariate analysis. Video recordings were evaluated, and scores were collected in a digital spreadsheet. All video files were subsequently deleted to protect participant privacy. Video evaluations were analysed as described above.

## 3 Results

A total of forty medical students have attended the ESMSC course, forming eight teams of five and going through both the low- and high-fidelity non-technical skill modules. The majority of students were in their final or penultimate year (57.5%) and the mean age was 23.15 (SD = 1.78). 70% of the students had previous teaching in the management of common surgical emergencies, while only 47.5% had teaching on non-technical skills (e.g. communication, team working, decision making).

### 3.1 Team performance video assessment

Participants’ NTS performance was higher in the low-fidelity intervention as assessed by the TEAM tool using the video recordings. This was true both for the mean of the 11 items of the questionnaire assessing leadership, teamwork and task management, and also for the global score (Table 1). The difference in the mean score between the low- and high-fidelity intervention was statistically significant, as analysed by a paired samples *t*-test (0.42, SD = 0.41, p = 0.023). However, this has not been the case for the global score, which regardless being higher by 0.63 for the low-fidelity intervention, it was no statistically significant (SD = 1.19, p = 0.18). When inspecting the individual items of the questionnaire, statistically significant changes were noted for items 2, 7, 8 and 9.

**Table 1:** Non-technical skill team performance as assessed using the TEAM tool.

	Non-technical skill	Low Fidelity		High Fidelity	
		n	Mean (standard deviation)	n	Mean (standard deviation)
1	The team leader let the team know what was expected of them through direction and command	8	2.38 (0.52)	8	2.50 (0.93)
2	The team leader maintained a global perspective	8	3.00 (0.76)	8	2.25 (0.46)
3	The team communicated effectively	8	2.75 (0.46)	8	3.00 (0.76)
4	The team worked together to complete the tasks in a timely manner	8	2.25 (0.46)	8	2.25 (0.71)
5	The team acted with composure and control	8	3.00 (0.00)	8	2.63 (0.52)
6	The team morale was positive	8	3.00 (0.53)	8	2.50 (0.76)
7	The team adapted to changing situations	8	2.75 (0.71)	8	1.63 (0.52)
8	The team monitored and reassessed the situation	8	2.75 (0.71)	8	1.63 (0.52)
9	The team anticipated potential actions	8	2.25 (0.46)	8	1.38 (0.52)
10	The team prioritised tasks	8	2.13 (0.35)	8	2.13 (0.83)
11	The team followed approved standards and guidelines	8	3.13 (0.35)	8	2.88 (0.35)
	**Overall (Items 1**–**11)**	**8**	**2.67 (0.32)**	**8**	**2.25 (0.14)**
	**Global score**	**8**	**6.88 (0.83)**	**8**	**6.25 (0.89)**

### 3.2 Self-assessed confidence towards non-technical skill attitudes

An overall mean increase in self-assessed confidence towards NTS attitudes was noted before participants having any intervention compared to after the low- and high-fidelity interventions (Table 2). Participants have assessed their NTS performance to be 0.34 out 5 (SD = 0.67, p = 0.002) higher in the low-fidelity intervention and 0.34 out of 5 (SD = 0.81, p = 0.12) higher in the high-fidelity intervention, compared to before the course. There was no difference in NTS self-assessed performance between the low- and high-fidelity interventions. When inspecting the individual items of the questionnaire, statistically significant changes were noted between the pre- and low-fidelity intervention for items 4, 7 and 8. Similarly, between the low- and high-fidelity intervention, statistically significant changes were noted for items 2, 4, and 8. A Wilcoxon signed-rank test was carried out, which was in line with the paired-samples *t*-test and, thus, not reported in this manuscript. A multivariate analysis demonstrated a statistically significant difference across all attitudes towards NTS between pre- and low-fidelity interventions [F(9,31) = 2.838, p = 0.015; Wilk's Λ = 0.548] and between pre- and high-fidelity interventions [F(9,31) = 3.796, p = 0.003; Wilk's Λ = 0.476].

**Table 2:** Self-assessed confidence towards non-technical skills attitudes before and after interventions.

	When working in a team, I feel confident (1 = Not Confident at all – 5 = Very Confident)		Before Intervention	Low Fidelity		High Fidelity	
		n	Mean (standard deviation)	n	Mean (standard deviation)	n	Mean (standard deviation)
1	Asking Questions for clarification	40	4.15 (0.736)	40	4.43 (0.712)	40	4.30 (0.966)
2	Speaking up when I have a concern	40	4.05 (0.677)	40	4.25 (0.809)	40	4.43 (0.813)
3	Challenging counterproductive behaviour in colleagues constructively, objectively and proportionately	40	3.50 (0.784)	40	3.73 (1.198)	40	3.73 (1.086)
4	Being open to feedback from all team members and willing to reflect on feedback about performance and behaviour and acknowledging any mistakes	40	4.13 (0.992)	40	4.58 (0.675)	40	4.63 (0.628)
5	Inviting opinions from those who have not voiced their view	40	4.23 (0.832)	40	4.48 (0.816)	40	4.25 (1.127)
6	Taking responsibility for my actions	40	4.30 (0.883)	40	4.58 (0.675)	40	4.60 (0.672)
7	Admitting having made a mistake	40	4.08 (0.797)	40	4.68 (0.730)	40	4.68 (0.656)
8	Reflecting on my own performance and what I could have done better	40	3.98 (0.733)	40	4.53 (0.716)	40	4.58 (0.594)
9	Contributing to team discussion about how to improve performance of the team	40	4.18 (0.931)	40	4.45 (0.714)	40	4.45 (0.783)
	**Overall**	**40**	**4.06 (0.53)**	**40**	**4.41 (0.59)**	**40**	**4.40 (0.59)**

### 3.3 Stress levels and presence scale

Reported mean stress levels across the 6 individual items of the questionnaire were higher for the high-fidelity module compared to the low-fidelity module (Table 3). Of the 40 students, 23 have rated their stress levels to be higher in the high-fidelity module, 13 to be lower, whereas 4 students saw no difference. A Wilcoxon signed-rank test determined that there was a statistically significant median increase in stress levels (0.33 out of 5) when students attended the high-fidelity module (1.92 out of 5) compared to the low-fidelity module (1.67 out of 5), z = −2.08, p = 0.038.

**Table 3:** Stress levels.

	Stress Experienced (1 = Total disagreement – 5 = Total agreement)	Low Fidelity		High Fidelity	
		n	Mean (standard deviation)	n	Mean (standard deviation)
1	I found it hard to make decisions	40	2.45 (1.22)	40	2.70 (0.97)
2	I worked up a sweat	40	1.67 (0.83)	40	2.18 (1.15)
3	My muscles were tensed	40	1.68 (0.97)	40	2.05 (1.20)
4	I felt my heart raising	40	2.02 (1.05)	40	2.52 (1.28)
5	My stomach got upset	40	1.45 (0.88)	40	1.55 (0.93)
6	My mouth got dry	40	1.30 (0.79)	40	1.45 (0.90)
	**Overall**	**40**	**1.76 (0.70)**	**40**	**2.07 (0.80)**

Reported mean presence scale values across the 4 individual items of the questionnaire were higher for the high-fidelity module compared to the low-fidelity module (Table 4). Of the 40 students, 31 have rated their presence scale to be higher in the high-fidelity module, 6 to be lower, whereas 3 students saw no difference. A Wilcoxon signed-rank test determined that there was a statistically significant median increase in presence (0.63 out of 5) when students attended the high-fidelity module (4.00 out of 5) compared to the low-fidelity module (3.25 out of 5), z = −3.687, p < 0 .0005.

**Table 4:** Presence scale.

	Presence scale (1: Not true– 5: Fully true)	Low Fidelity		High Fidelity	
		n	Mean (standard deviation)	n	Mean (standard deviation)
1	I felt I was part of an actual team in a clinical setting	40	3.47 (0.987)	40	4.30 (0.853)
2	The scenario triggered my emotions (anger, sadness, satisfaction)	40	2.28 (1.132)	40	3.00 (1.485)
3	While managing the simulated patients, I forgot for the time being that I was taking part in a study	40	3.43 (1.259)	40	4.00 (1.281)
4	While managing the simulated patients, my thoughts became emerged in the clinical scenario	40	3.65 (1.272)	40	4.00 (1.281)
	**Overall**	**40**	**3.21 (0.725)**	**40**	**3.83 (0.915)**

### 3.4 Module evaluation

Evaluation scores for all individual items of the questionnaire were ≥4.60 in both NTS modules (Table 5). Students have assessed the high-fidelity module higher (4.88 out of 5, SD = 0.29) compared to low-fidelity module (4.74 out of 5, SD = 0.67). A paired-samples *t*-test demonstrated a statistically significant difference (0.14 out of 5, SD = 0.37, p = 0.020) between evaluations of the two modules. When inspecting the individual items of the questionnaire, a positive difference between the low- and high-fidelity intervention was noted in 13 out of 14 items of the questionnaire.

**Table 5:** Low- and high-fidelity module evaluations.

		Low Fidelity		High Fidelity	
		n	Mean (standard deviation)	n	Mean (standard deviation)
1	Overall, I liked this training	40	4.80 (0.464)	40	4.95 (0.221)
2	The learning atmosphere was pleasant	40	4.60 (0.709)	40	4.90 (0.379)
3	The learning was fun	40	4.65 (0.533)	40	4.85 (0.427)
4	I find the training useful for my degree	40	4.75 (0.679)	40	4.88 (0.404)
5	Investing time in this training was useful	40	4.75 (0.588)	40	4.95 (0.221)
6	I can apply the content of this training in my degree	40	4.65 (0.622)	40	4.95 (0.221)
7	I derive personal use from this training	40	4.65 (0.622)	40	4.90 (0.379)
8	The content was comprehensible	40	4.88 (0.335)	40	4.85 (0.533)
9	The language (foreign words and technical terms) was comprehensible	40	4.83 (0.446)	40	4.97 (0.158)
10	I kept up thematically in the training	40	4.75 (0.543)	40	4.83 (0.501)
11	The time was sufficient for the themes covered	40	4.58 (0.813)	40	4.63 (0.868)
12	I will apply what I leaned to my day-to-day work	40	4.78 (0.480)	40	4.88 (0.335)
13	I find it good that I received feedback after each session	40	4.85 (0.427)	40	4.88 (0.404)
14	I would recommend this training to my colleagues	40	4.93 (0.350)	40	4.97 (0.158)
	**OVERALL**	**40**	**4.74 (0.393)**	**40**	**4.88 (0.290)**

## 4 Discussion

To this point, there are no established undergraduate team-performance training courses, neither incorporated in medical school curricula, nor developed by professional organisations [8]. Nevertheless, a recent systematic review has identified 68 studies from various institutions globally, describing courses that target a concoction of NTS; however, the consensus was that such efforts lack a standardised structure, both in their intervention and evaluation approaches [8]. In this study, we have described a novel team-performance training course for medical students in an attempt to explore its effectiveness and also establish a beneficial fidelity level for such approaches.

### 4.1 Presence

Presence, in the context of medical simulation training, relates to the extent to which the user feels part of the virtual experience; in theory, maximum presence is reached when the user can no longer differentiate between real and replicated [24,25]. The items of the presence scale questionnaire used in our study were extracted from Witmer & Singer's validated questionnaire and modified accordingly [24]. Based on our results, it can be deduced that students felt more included in their teams in the high-fidelity module when compared to the low-fidelity one. Not only did it triggered their emotions more, their thoughts also became further emerged in the clinical scenario and they even, momentarily, forgot that they were taking part in a study (Table 4). Arguably, such experiences can be sparked more strongly in a high-fidelity simulation environment, where participants come across realistic case scenarios, with a dubious line separating reality and replication [26]. Evidently, higher levels of ‘presence’ seem to correlate to higher levels of fidelity [24,27,28]. Assuming that this is true, our results demonstrate an obvious difference in fidelity between the two interventions used; a distinction which is objective and, thus, allows for other factors such as team performance to be compared reliably.

### 4.2 Team performance

Self-assessed, subjective confidence towards NTS attitudes has been significantly improved, both after the low- and high-fidelity interventions, when compared to having no intervention at all (Table 2). Such positive improvement can be attributed to the pre-intervention training on the A-E approach, to the structured feedback provided and teaching after the mock attempt. It has previously been suggested that expert-feedback can indeed improve performance in NTS; this comes in line with our findings [29]. However, Stefanidis et al. concluded that expert feedback should be kept at a minimum to accelerate learning [30]. Our results seem to come in line with the aforementioned conclusion, since only a 20-min period was allocated for feedback and discussion for the whole group. Furthermore, participants were allowed adequate time to reflect on errors they made, both through a constructive discussion led by the facilitator, and also in their own time. Self-reflection techniques are currently used in medical schools and required by the General Medical Council [31]. Moreover, regardless of this being a subjective assessment, it has nonetheless demonstrated a substantial increase in participant confidence concerning fundamental NTS such as communication, teamwork, decision making and self-reflection. Arora et al. support that non-technical skills should be assessed formally by objective expert input, as novice surgeons lack insight into their behaviours [32]. On the contrary, we propose that participant confidence in such attitudes is equally important as objective third-party assessments. This is further supported by Bosch & Mansell, who suggest that confidence is a prerequisite in building team trust, consequently enhancing team performance [33].

Objective team performance was assessed using the TEAM tool, a previously validated and reliable tool used in emergency scenarios [34,35]. Cant et al. used the tool to assess the NTS of hospital medical emergency teams and concluded that ‘*it is a valid, reliable and easy to use tool, for both training and clinical settings, with benefits for team performance when used as an assessment and/or debriefing tool*’. The psychometric properties of the tool were also validated in simulated scenarios for resuscitation at the University of Grenoble in France [36]. The objective team performance results of our study demonstrate a significantly higher performance in the low-fidelity intervention compared to the high-fidelity one, whereas subjective self-assessment of NTS attitudes showed no difference (Table 1). This comes in line with a literature review by Munshi et al., demonstrating that high fidelity levels of simulation do not necessarily yield better results [18]. Moreover, a study by Massoth et al. on the Advanced Life Support skills and knowledge of medical students, demonstrated not only that high-fidelity simulation led to equal or inferior performance, but it also resulted in overconfidence [20]. In practical skill simulation, it has been speculated that levels of fidelity should change accordingly to the type of task and level of trainee, and that minimal resources should be utilised for a given level of performance [18,37,38]. Based on our results, such conclusions are potentially true for non-practical skills as well. Our course, targeting medical students – the most junior medical trainees – should have been centred around low-fidelity intervention modalities matching the undergraduate level of knowledge. Nevertheless, we support that low- and high-fidelity interventions should be used in harmony; the former for development of basic soft skills and the latter to challenge the participants and refine the taught skills.

### 4.3 Stress

The inferior level of team performance noted in the high-fidelity intervention, can be potentially attributed to the stress experienced by the students. Studies have repeatedly concluded that stress is indeed a negative factor for performance in various skills in simulation settings [[39], [40], [41]]. A randomised crossover study in Netherlands showed that NTS performance declined when external stressors were induced to the simulated scenarios [41]. Similarly, in our study, participants rated the high-fidelity intervention to be more stressful in all six items of the questionnaire (Table 3). This can be attributed to the use of a simulated patient wearing the hyper-realistic suit, which was bleeding, breathing and experiencing pain. Andreatta et al. argued that the induced stress during simulation-based training can indeed be beneficial to the surgical novice, as it might harvest stress management skills that are essential in the applied clinical setting [42]. Nevertheless, stress levels for both modules were rated to be lower than 2.1 out of 5, suggesting that regardless of the difference between them, the overall environment was not very stressful for the participants.

### 4.4 Limitations

Limitations of our study include organising the intervention at a single institution with a rather small sample size. Furthermore, due to the fact that students were inevitably prompted by the facilitator during the mock attempt, objective assessment using the TEAM tool was deemed impractical. Future studies could introduce activities that require no prompting by the faculty and thus allowing a robust objective performance improvement in NTS.

### 4.5 Evaluation

The evaluations of both the low- and high-fidelity interventions were exceedingly positive, as all items of the questionnaire attained a score greater than 4.6 out of 5 (Table 5). Participants found this activity pleasant, valuable to their degree and they would recommend it to their colleagues. This is a particularly valuable outcome of our study as it signals the possibility for further development and, most importantly, suggests successful implementation of such interventions in medical school curricula. There are currently no established methods of assessing team performance of medical students in undergraduate training.

One can argue that soft skills are indeed taught through clinical rotations and clinical skill training; however, such efforts are mainly targeted at improving the personal performance of the student [43]. This is further supported by the fact that clinical skill assessment is largely based on individual tasks through Objective Structured Clinical Examination (OSCE) stations, which disregard the medical student's ability to perform as part of a team [44]. Such practice neglects the organisational structure of today's healthcare systems and potentially falsely nurtures individuality in medical graduates. We suggest that interventions aiming to improve NTS should be implemented in the medical school curricula and, moreover, that the taught skills, and particularly team performance, should be assessed formally using validated tools such as TEAM. Such approaches can make the transition from medical school to the clinical environment smoother, by yielding health care professionals of heightened inter-professional abilities, who are able to adapt to the multifaceted social environments created by globalised medicine and ever-growing public pressures.

## 5 Conclusions

Both the low- and high-fidelity interventions integrated in the three-day surgical course, demonstrated an improvement in the team performance abilities of attending medical students. The high-fidelity intervention was indeed more realistic, yet more stressful for the participants. Furthermore, it proved to be superior in harvesting leadership, teamwork and task management skills. Both modules were evaluated highly by the students, however, future research should address retention of the taught skills and adaptability of such interventions in medical school curricula.

## Ethical approval

The ESMSC course is compatible with the current 3R principles for animal-model simulation (refinement, replacement and reduction). Ethical approval was granted and met directive 63/2010, PD 56/April 2013, according to the local ethics committee. The license reference number is 4857/15-09-2017, MS, AP et al. All procedures performed in studies involving human participants were in accordance with the ethical standards of the institutional and/or national research committee and with the 1964 Helsinki declaration and its later amendments or comparable ethical standards. All applicable international, national, and/or institutional guidelines for the care and use of animals were followed.

## Sources of funding for your research

Those ESMSC course series were funded at the time by the Experimental Educational and Research Centre ELPEN.

## Author contribution

Please specify the contribution of each author to the paper, e.g. study concept or design, data collection, data analysis or interpretation, writing the paper, others, who have contributed in other ways should be listed as contributors.

Contributorship Statement.

• M.N. has contributed in the conception and design of the work, data collection, data analysis and interpretation, drafting the article and final approval of the version to be published.
• E.T. has contributed in data collection, drafting the article and final approval of the version to be published. E.T. is equal contributor with E.I.E.
• E.I.E. has contributed in data collection, drafting the article and final approval of the version to be published. E.I.E. is equal contributor with E.T.
• I.T. has contributed in drafting the article and final approval of the version to be published.
• N.A. has contributed in the conception and design of the work and final approval of the version to be published.
• N.L. has contributed in drafting the article and final approval of the version to be published.
• F.O. has contributed in drafting the article and final approval of the version to be published.
• D.K. has contributed in the design of the work and final approval of the version to be published.
• E.A. has contributed in the design of the work and final approval of the version to be published.
• A.P. has contributed in the conception and design of the work and final approval of the version to be published. A.P. is equal contributor with M.S.
• M.S. has contributed in the conception and design of the work, data interpretation, drafting the article and final approval of the version to be published. M.S. is equal contributor with A.P.

## Guarantor

Michail Sideris is the guarantor of this work.

## Consent

No patients or volunteers consent needed – Ethical approval of this study was under the same framework for the ESMSC Course.

## Provenance and peer review

Not commissioned externally peer reviewed.

## Registration of research studies

Researchregistry5617.

https://www.researchregistry.com/browse-the-registry#home/?view_2_search=Michail%20Sideris%20&view_2_page=1.

## Declaration of competing interest

None declared.
